# Drug-likeness analysis of traditional Chinese medicines: 1. property distributions of drug-like compounds, non-drug-like compounds and natural compounds from traditional Chinese medicines

**DOI:** 10.1186/1758-2946-4-31

**Published:** 2012-11-27

**Authors:** Mingyun Shen, Sheng Tian, Youyong Li, Qian Li, Xiaojie Xu, Junmei Wang, Tingjun Hou

**Affiliations:** 1 Institute of Functional Nano & Soft Materials (FUNSOM) and Jiangsu Key Laboratory for Carbon-Based Functional Materials & Devices, Soochow University, Suzhou, Jiangsu, 215123, China; 2 College of Pharmaceutical Sciences, Soochow University, Suzhou, Jiangsu, 215123, China; 3 Department of Biochemistry, The University of Texas Southwestern Medical Center, 5323 Harry Hines Blvd, Dallas, TX, 75390, USA; 4 College of Chemistry and Molecular Engineering, Peking University, Beijing, 100871, China

**Keywords:** Drug-likeness, Traditional Chinese medicines, Principal component analysis (PCA), Property distribution, Molecular properties

## Abstract

**Background:**

In this work, we analyzed and compared the distribution profiles of a wide variety of molecular properties for three compound classes: drug-like compounds in MDL Drug Data Report (MDDR), non-drug-like compounds in Available Chemical Directory (ACD), and natural compounds in Traditional Chinese Medicine Compound Database (TCMCD).

**Results:**

The comparison of the property distributions suggests that, when all compounds in MDDR, ACD and TCMCD with molecular weight lower than 600 were used, MDDR and ACD are substantially different while TCMCD is much more similar to MDDR than ACD. However, when the three subsets of ACD, MDDR and TCMCD with similar molecular weight distributions were examined, the distribution profiles of the representative physicochemical properties for MDDR and ACD do not differ significantly anymore, suggesting that after the dependence of molecular weight is removed drug-like and non-drug-like molecules cannot be effectively distinguished by simple property-based filters; however, the distribution profiles of several physicochemical properties for TCMCD are obviously different from those for MDDR and ACD. Then, the performance of each molecular property on predicting drug-likeness was evaluated. No single molecular property shows good performance to discriminate between drug-like and non-drug-like molecules. Compared with the other descriptors, fractional negative accessible surface area (FASA-) performs the best. Finally, a PCA-based scheme was used to visually characterize the spatial distributions of the three classes of compounds with similar molecular weight distributions.

**Conclusion:**

If FASA- was used as a drug-likeness filter, more than 80% molecules in TCMCD were predicted to be drug-like. Moreover, the principal component plots show that natural compounds in TCMCD have different and even more diverse distributions than either drug-like compounds in MDDR or non-drug-like compounds in ACD.

## Background

The development of high-throughput screening (HTS) technique brings increased capability for screening large number of compounds against relevant biological targets in a relatively short period of time [1]. In order to meet the increasing demand of HTS many chemical companies tried to collect extensive small molecule libraries commercially available for medicinal chemists. In the latest version of Available Chemicals Directory (ACD) database, over 3,870,000 unique chemicals can be purchased from 901 suppliers. However, it is not a wise strategy to purchase all these commercial available molecules for HTS, as only a small fraction of compounds in these commercial available databases are drug-like. Therefore, in the first step of drug discovery it is quite necessary to apply some drug-like filters to eliminate the non-drug-like molecules from the databases and then focus on drug-like molecules only. Nowadays, drug-likeness evaluation (e.g. the Lipinski’s Rule-of-Five [2], the Opera’s rules of drug-likeness [3], the ROES filter [4], etc.) has already been, to some extent, integrated into the computational drug design/discovery pipelines. In the last decades, substantial efforts have been made in the development of computational approaches for differentiating drug-like molecules from reagents, such as the simple property-based filters or rules [2-8], the drug-like index to rank molecules [9,10], the characterization of molecular frameworks and side chains [11-13], the classification models of drug-likeness based on decision trees (DTs), artificial neural networks (ANNs), support vector machines (SVMs), etc. [14-18].

Traditional medicines, especially herbal or botanic medicines, are very important in health care systems around the world. According to the statistics from the World Health Organization (WHO), in many Asian and African countries, 80% of the population depends on traditional medicines for primary health care. Herbal treatments are the most popular form of traditional medicines. In China, traditional Chinese medicines (TCMs) have been developed for therapeutic use for more than 4000 years. The classic TCMs are primarily based on a large number of herbal formulations that are used for the treatment of a wide variety of diseases. It is believed that TCMs are a rich source of therapeutic leads for the pharmaceutical industry [19]. TCMs are gaining more and more attention in clinical practices throughout the world [20,21]. Considering the dominant role of natural products in the discovery and development of drugs for the treatment of human diseases, discovery of new bioactive compounds from herbs used in TCMs and identification of their pharmacological effects are becoming a promising way for finding new drugs [22]. Certainly, the path from traditional Chinese medicines to Western pharmaceuticals is fraught with challenges, including isolation and identification of active components or compounds, elucidation of pharmaceutical mechanism, and development as a pharmaceutical.

Previous studies showed that compounds from the herbs used in TCMs may be a good source for drug discovery after being evaluated by drug-likeness filters [6]. However, the in-depth analysis of the structural features and the drug-likeness evaluation of compounds identified in TCMs are still lacking. To give relatively accurate evaluation of the structural features and drug-likeness for TCMs, the following two requirements need to be satisfied: first of all, reliable drug-like filters, reasonable schemes to characterize structural features and accurate prediction models of drug-likeness are necessary; second, the number of compounds from TCMs should be enough in order to guarantee the statistical robustness of drug-likeness analysis. Recently, the Traditional Chinese Medicine Compound Database (TCMCD) developed in our group has been updated and the latest version contains more than 60,000 unique compounds [23]. To our knowledge, the number of the molecules in TCMCD is larger than those in several other similar databases developed in other groups [24-26]. We believe that based on the extensive data in TCMCD reliable results and conclusions can be guaranteed.

In this paper series, we set out to investigate the property distributions, characterize the structural features and evaluate the drug-likeness of molecules in TCMCD systematically, which include (1) the analysis and comparison of the property distributions for ACD, MDDR and TCMCD, (2) the characterization and comparison of the scaffold architectures for ACD, MDDR and TCMCD, and (3) the quantitative evaluation of drug-likeness for TCMCD based on the classification models of drug-likeness [27]. There are two objectives of this series. First of all, we hope to examine and establish a set of computational strategies, including the property-based rules, the characterization of molecular frameworks and the classification models based on naïve Bayesian classification technique, for drug-likeness evaluation at different levels. Second, we try to conduct in-depth drug-likeness analysis for TCMCD using different strategies. We expect that our studies can guide pharmaceutical scientists to promote the development of TCMs.

In the first paper of this series, we focused on the analysis of the property distributions for ACD, MDDR and TCMCD, and then compared the performance of the simple property-based filters of drug-likeness and evaluated the drug-likeness of TCMCD by using these filters. Compared with property-based filters, the classification models of drug-likeness based on machine learning techniques can give more accurate predictions. However, in drug design process, the property-based filters, such as the Lipinski’s Rule-of-Five [2] and the Opera’s rules of drug-likeness [3], are much more popular than the more complicated classification models because they can be easily understood and utilized by scientists besides the computational chemists. It should be noted that the property-based filters developed from different databases are not always consistent and the prediction accuracies of some filters are not reliable. Therefore, in this study, we not only just used the popular filters but also evaluated the new filters based on different molecular properties.

## Methods

### Preparation of the datasets

Here, the MDDR and ACD databases were chosen as representatives for drug-like and non-drug-like datasets, respectively. The Traditional Chinese Medicine Compound Database (TCMCD) was developed in our group [23,28]. The latest version of TCMCD has 63,759 organic molecules identified from more than 5,000 herbs in TCMs. All the molecules in these three databases were minimized in MOE [29] by using molecular mechanics (MM) with the MMFF94 force field [30]. The three databases were preprocessed using the following protocol [6,31,32]: (1). Molecules were examined for bad valence states, and molecules containing one or more atoms with bad valence states were removed; (2). The salt fragments in the input molecules were identified and removed; (3). The molecules with atoms other than C, H, O, N, P, S, F, Cl, Br and I were removed; (4). The solvent molecules in the input molecules were identified and removed; (5). The input molecules with multiple organic parts were identified and the largest connected structural fragment in each input molecule was reserved; (6). Duplicates were removed in each individual database; (7). Identical compounds found in both ACD and MDDR databases were removed from ACD. For MDDR, antineoplastic drugs were removed because they are often highly cytotoxic and are likely to react with protein targets. In addition, the compounds (adsorption promoters, anesthetics, diagnostic agents (isotope), diagnostics for AIDS, diagnostics for cancer, drug delivery systems, magnetic resonance imaging agents, sweeteners, and dental agents) without therapeutic activity were eliminated from MDDR. As a result, we got 2,175,382 molecules from ACD, 142,747 molecules from MDDR and 63,759 molecules from TCMCD for the following analysis.

It should be noted that we did not remove compounds with reactive functional groups. We did a survey on how many reactive compounds in three data sets. A simple filter was designed to remove compounds with reactive functional groups, and the reactive functional groups used by us include aldehyde, alkyl-halide, anhydride, diazo, dicarbonyl, disulfide, hydrazine-N-NH2, isocyanate, isothiocyanates, peroxide, quaternaryamine and acyl-halide.[6] When this filter was applied, ~5% of compounds in MDDR were removed as reactive molecules; however, ~6% of launched drugs in MDDR were also removed as reactive molecules. Moreover, based on Opera’s analysis, removing reactive compounds from ACD and MDDR does not have obvious impact on the performance of the drug-likeness filters [3], so we did not remove the molecules with reactive functional groups.

It is well-known that too large molecules usually do not have good absorption property [33,34], and therefore we set the cutoff for molecular weight (MW) to be 600, and the sub-databases, namely ACD1, MDDR1 and TCMCD1, respectively, were constructed by only choosing the molecules with MW less than 600. Furthermore, to examine the influence of the MW cutoff on our analysis, three more subsets (ACD2, MDDR2 and TCMCD2) with MW less than 800 were generated. The numbers of the compounds in MDDR1, ACD1 and TCMCD1 are 123,927, 1,999,530 and 50,962, respectively, and the numbers of the compounds in MDDR2, ACD2 and TCMCD2 are 138,507, 2,007,594 and 57,809, respectively.

Comparison study showed that the mean MW of compounds in ACD1 was about 120 less than that of compounds in MDDR1. It is believed that many molecular properties are dependent on MW, and so in order to construct the filters or models of drug-likeness unrelated to MW, a subset of ACD1 designated as ACD3 and a subset of TCMCD1 labeled as TCMCD3 were constructed, and ACD3, TCMCD3 and MDDR1 have almost the same MW distributions. In total, there are 123,927 123,929 and 33,961 entries in ACD3, MDDR1 and TCMCD3, respectively. For the purpose of performing principal component analysis (PCA), the same number of entries as that of TCMCD3 (33,961) were randomly selected from MDDR1 and ACD3 to form another two subsets, MDDR3 and ACD4, respectively.

### Calculations of molecular descriptors

In the current study, 44 molecular descriptors were used, including octanol-water partitioning coefficient (Alog*P*) based on the Ghose and Crippen's method [7], the apparent partition coefficient at pH = 7.4 (log*D*_7.4_) based on the Csizmadia’s method [35], molecular solubility (log*S*) based on the multiple linear regression model developed by Tetko et al. [36], MW, the number of hydrogen bond donors (*n*_HBD_), the number of hydrogen bond acceptors (*n*_HBA_), the number of rotatable bonds (*n*_rot_), polar surface area (PSA), the number of hydrogen bond donors used by Lipinski’s Rule-of-Five (*n*_HBDL_), the number of hydrogen bond acceptors used by Lipinski’s Rule-of-Five (*n*_HBAL_), molecular surface area (MSA), the number of carbon atoms (*N*_c_), the number of nitrogen atoms (*N*_n_), the number of oxygen atoms (*N*_o_), the number of halogens (*N*_halogen_), 23 descriptors to count atoms, bonds and rings (descriptors 16 ~ 38), and 6 Kier & Hall subgraph count index (descriptors 39 ~ 44) [37]. All the descriptors were calculated using Discovery Studio molecular simulation package (version 2.5) [38]. The descriptions of the descriptors are summarized in Additional file 1: Table S1 in the supporting materials.

### Performance of each molecular property for drug-likeness evaluation

The performance of each property shown in Additional file 1: Table S1 for distinguishing ACD from MDDR was evaluated, and the best value for classification was determined by a grid search. It should be noted that many descriptors in Additional file 1: Table S1 are molecular size dependent. It is obvious that larger compounds bear, on average, more functional groups, which may produce more hydrogen bond donors, hydrogen bond acceptors, flexible bonds, ring systems, and larger polar surface area. It is therefore understandable that the drug-likeness filters based on these molecular descriptors are database dependent because the distributions of the size-related descriptors are different from a database to another. In order to develop drug-likeness filters that are database independent for distinguishing drug-like from non-drug-like molecules, the size-unrelated descriptors are preferred. Here, 16 molecular descriptors based on the ratio of different molecular properties (Additional file 1: Table S2 in the supporting materials) were examined. These descriptors in Additional file 1: Table S2 include fraction of rotatable bonds (*f*_rot_), fraction of polar surface area (*f*_PSA_), six fractional descriptors for water accessible surface, six fractional descriptors for van der Waals surface, C3P, and UNC_C3. The twelve fractional descriptors for water accessible surface and van der Waals surface were calculated using MOE molecular simulation package [29]. The descriptors, C3P, and UNC_C3, were proposed by Zheng and coworkers [6]. The descriptor C3P is the ratio of the number of sp3 hybridized C atoms to the number of the total heavy atoms except halogen atoms; and the descriptor UNC_C3 represents the ratio of the number of unsaturated carbon atoms to the number of sp3 carbon atoms and is believed to be related to molecular saturation and rigidity. The quality of each descriptor for distinguishing ACD from MDDR was measured by the quantity of true positives (*TP*), true negatives (*TN*), false positives (*FP*), false negatives (*FN*), sensitivity (*SE*), and specificity (*SP*), the prediction accuracy for MDDR molecules (*PRE*1), the prediction accuracy for ACD molecules (*PRE*2), the global accuracy (*GA*), and the Matthews correlation coefficient (*C*):

(1) $$SE=\frac{TP}{TP+FN}$$

(2) $$SP=\frac{TN}{TN+FP}$$

(3) $$PRE1=\frac{TN}{TN+FP}$$

(4) $$PRE2=\frac{TN}{TN+FN}$$

(5) $$GA=\frac{TP+TN}{TP+TN+FP+FN}$$

(6) $$C=\frac{TPxTN-FNxFP}{\sqrt{\left(TP+FN\right)\left(TP+FP\right)\left(TN+FN\right)\left(TN+FP\right)}}$$

*GA* and *C* are two important indicators for classification accuracy.

### Principal component analysis (PCA) for ACD, MDDR and TCMCD

The principal component analysis was performed using the MOE molecular simulation package. All descriptors listed in Additional file 1: Tables S1 and S2 were included in PCA. The PCA were performed for the three subsets (ACD4, MDDR3 and TCMCD3) that have the same number of entries (33,961). Because ACD3, MDDR1 and TCMCD3 were constructed to have similar MW distributions, the three subsets in PCA also have similar MW distributions. To ensure that the selected subsets were representative, the means of important molecular properties were calculated for these randomly generated subsets (ACD4, MDDR3 and TCMCD3). All were found to be almost as the same as the values for the complete datasets (ACD3, MDDR1 and TCMCD3).

## Results and discussion

### Distributions of important properties for ACD1, MDDR1 and TCMCD1

**Figure 1:**
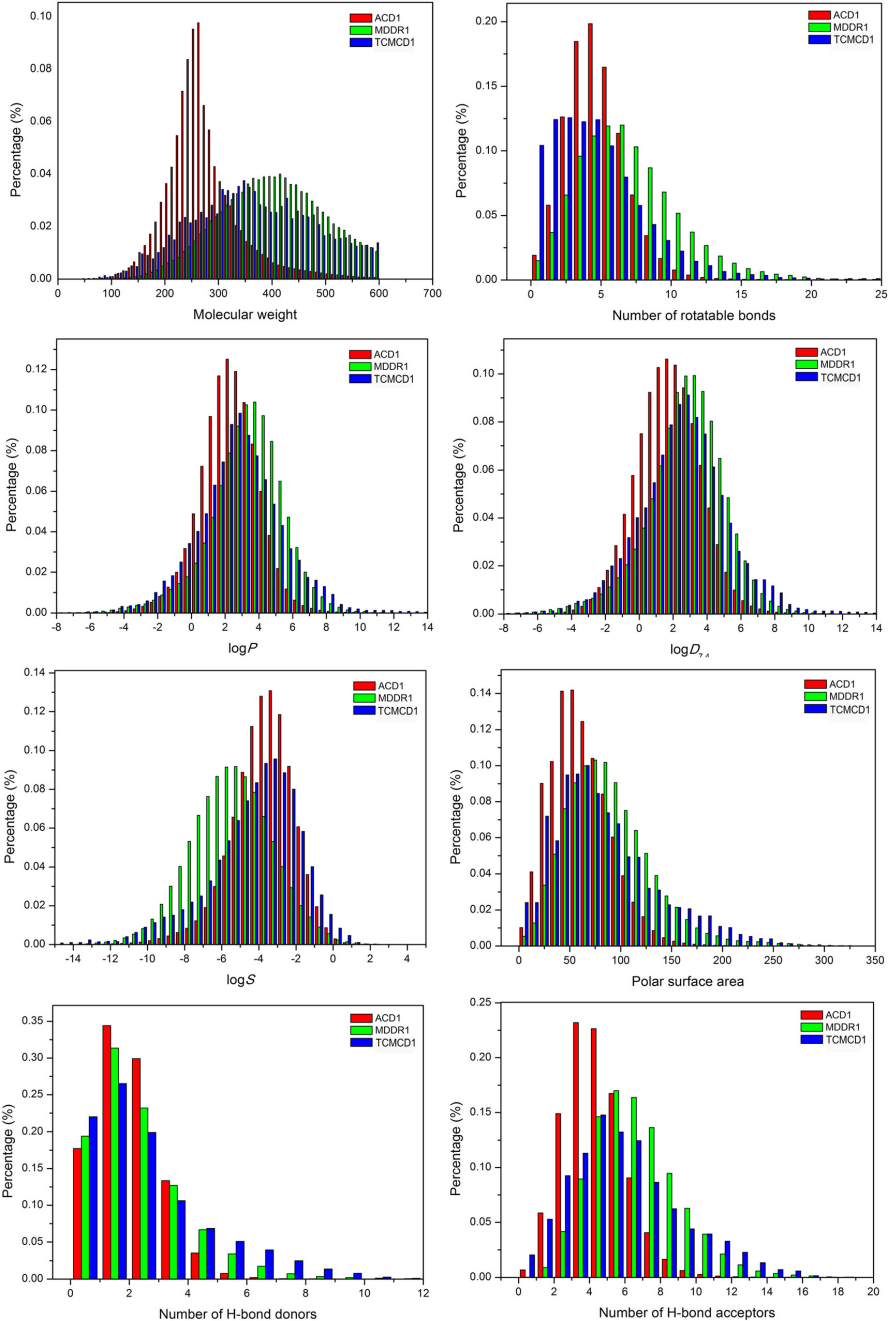
The distributions of eight important molecular property descriptors for ACD1, MDDR1 and TCMCD1.

First, we performed profile analysis of eight important molecular properties for all molecules with MW smaller than 600 extracted from ACD, MDDR and TCMCD (ACD1, MDDR1 and TCMCD1). These eight important molecular properties, include log*P*, log*D*_7.4_, log*S*, MW, *n*_rot_, PSA, *n*_HBD_ and *n*_HBA_, are widely used in drug-likeness analysis and ADME predictions [33,34,39-42]. The distributions of these molecular properties for ACD1, MDDR1 and TCMCD1 are shown in Figure 1. The mean values of all descriptors listed in Additional file 1: Table S1 are displayed in Table 1.

**Table 1:** The mean values of different properties for ACD, MDDR and TCMCD

**No.**	**Descriptors**	**ACD**			**MDDR**		**TCMCD**		
		**1**	**2**	**3**	**1**	**2**	**1**	**2**	**3**
		<600	<800	<600	<600	<800	<600	<800	<600
1	Alog*P*	2.29	2.30	3.84	3.24	3.30	2.84	2.73	3.02
2	log*D*_7.4_	1.78	1.79	3.45	2.71	2.77	2.53	2.41	2.71
3	log*S*	−3.76	−3.79	−6.00	−5.51	−5.76	−4.34	−4.41	−4.71
4	MW	270	272	398	400	425	366	403	400
5	*N*_HBA_	3.22	3.24	4.41	4.83	5.22	5.27	6.32	5.82
6	*N*_HBD_	1.25	1.26	1.24	1.64	1.82	2.12	2.72	2.33
7	*N*_rot_	4.28	4.32	6.08	6.45	7.02	4.44	5.08	4.88
8	PSA	61.4	61.8	81.3	88.2	95.4	84.5	99.7	92.4
9	*N*_HBAL_	3.88	3.90	5.33	6.02	6.51	5.50	6.48	6.04
10	*N*_HBDL_	1.54	1.55	1.39	1.83	2.01	2.14	2.74	2.35
11	MSA	262	264	365	382	406	358	394	390
12	*N*_C_	13.5	13.6	19.6	21.2	22.4	20.3	22.4	22.2
13	*N*_N_	1.94	1.94	2.30	2.89	3.03	0.477	0.300	0.459
14	*N*_O_	1.94	1.96	3.03	3.12	3.48	5.03	6.18	5.59
15	*N*_Halogen_	0.655	0.658	1.12	0.647	0.664	0.139	0.0130	0.154
16	*N*_Atom_	18.3	18.4	26.5	28.2	29.9	26.1	28.9	28.5
17	*N*_Bonds_	19.2	19.3	28.5	30.6	32.5	28.3	31.5	31.0
18	*N*_positive_	0.0478	0.048	0.0706	0.0519	0.056	0.0348	0.017	0.0347
19	*N*_negative_	0.0508	0.052	0.0797	0.0696	0.074	0.0341	0.012	0.0341
20	*N*_Spiro_	0.00428	0.0040	0.0101	0.0200	0.021	0.0388	0.042	0.0459
21	*N*_BHA_	0.0229	0.0240	0.0355	0.106	0.121	0.456	0.542	0.503
22	*N*_Ringb_	10.6	10.7	16.8	18.6	19.3	16.9	18.7	18.5
23	*N*_aromatic_	8.16	8.19	13.0	12.4	12.7	5.79	6.01	6.07
24	*N*_Bridge_	0.0880	0.0930	0.139	0.456	0.555	1.75	2.13	1.92
25	*N*_Rings_	1.92	1.93	3.00	3.41	3.52	3.27	3.59	3.58
26	*N*_AR_	1.43	1.44	2.27	2.19	2.25	0.999	1.03	1.05
27	*N*_RA_	1.65	1.66	2.43	2.52	2.59	1.51	1.67	1.61
28	*N*_R3_	0.0407	0.041	0.0198	0.0401	0.043	0.0770	0.0721	0.0823
29	*N*_R4_	0.00864	0.0090	0.00635	0.0427	0.044	0.0111	0.0120	0.0103
30	*N*_R5_	0.474	0.475	0.644	0.815	0.825	0.705	0.696	0.762
31	*N*_R6_	1.38	1.38	2.31	2.44	2.53	2.35	2.68	2.59
32	*N*_R7_	0.0154	0.015	0.0193	0.0625	0.062	0.0693	0.064	0.0735
33	*N*_R8_	0.00202	0.0020	0.00148	0.00356	0.0040	0.0128	0.020	0.0152
34	*N*_R9+_	6.21E-4	8.27E-4	0.00285	0.00620	0.018	0.0425	0.043	0.0446
35	*N*_Chains_	21.0	21.1	26.7	30.6	32.7	33.3	37.3	36.5
36	*N*_ChainA_	8.51	8.55	12.7	13.5	14.0	12.0	13.3	13.1
37	*N*_Stereo_	0.550	0.561	0.720	1.25	1.49	4.14	5.12	4.76
38	*N*_StereoB_	0.163	0.166	0.442	0.493	0.524	1.06	1.09	1.13
39	SC0	18.3	18.4	26.5	28.2	29.9	26.0	28.9	28.4
40	SC1	19.2	19.3	28.5	30.6	32.5	28.3	31.5	31.0
41	SC2	26.1	26.3	40.0	43.2	45.9	42.9	48.1	47.4
42	SC3P	32.0	32.3	51.5	56.5	60.0	60.0	67.8	66.9
43	SC3C	6.20	6.25	9.88	10.7	11.5	13.6	15.4	15.4
44	SC3CH	0.0407	0.041	0.0198	0.0401	0.043	0.0770	0.072	0.0823

There is remarkable difference in the distributions between ACD1 and MDDR1. The mean values of MW for ACD1 and MDDR1 molecules are 270 ± 68.8 and 400 ± 95.5, respectively. The mean value of MW for TCMCD1 molecules is 366 ± 113.6, which is quite close to that of drug-like molecules in MDDR1 while significantly higher than that of non-drug-like molecules in ACD1. As shown in Figure 1, the MW distribution of TCMCD1 is highly overlapped with that of MDDR1 and slightly skewed toward lower molecular weights. Feher and co-workers also observed that the MW distribution of natural compounds peaks at a similar position as drugs. However, after inspecting the results reported by Feher et al. [8], we found that our results were quite different from the previous observations. In Feher’s work, the MW distributions of drugs and natural compounds are quite different although they peak at a similar position. According to the Feher’s results, the mean molecular weights for drugs and natural compounds are 340 and 414, respectively, which are obviously different from those shown in Table 1. In Feher’s analysis, the numbers of drugs and natural compounds are quite limited (10,968 and 3278, respectively), which might not guarantee the reliability of their results.

For the molecular descriptors studied here, three of them are related to hydrophobicity of a molecule: log*P*, log*D*_7.4_ and log*S*. The distributions of these three descriptors for ACD1, MDDR1 and TCMCD1 are shown in Figure 1. The mean values of log*P* for MDDR1 *versus* ACD1 are 3.24 and 2.29, respectively, which reflects the fact that the mean MW of ACD1 is much smaller than those of MDDR1 and TCMCD1 since small molecules are usually less hydrophobic. The average values of log*D*_7.4_ for MDDR1 *versus* ACD1 are 2.71 and 1.78, respectively. As shown in Figure 1 and Table 1, molecules in TCMCD1 (the mean values of log*P* and log*D*_7.4_ for TCMCD1 are 2.84 and 2.53, respectively) are a little more lipophilic than drug-like molecules in MDDR1 but much more hydrophobic than non-drug-like molecules in ACD1. To summarize, the distributions of log*P* and log*D*_7.4_ of TCMCD1 are closer to those of MDDR1 than those of ACD1.

The calculated mean values of log*S* for MDDR1, ACD1 and TCMCD1 are around −5.51, -3.76 and −4.34, respectively. From the distributions of log*S* for MDDR1 and ACD1 shown in Figure 1, we conclude that log*S* is a better descriptor than log*P* and log*D*_7.4_ to discriminate ACD1 from MDDR1. Obviously, drug-like molecules in MDDR1 show an obvious tendency towards decreased solubility. Overall, molecules in TCMCD1 are more soluble than those in MDDR1 while less soluble than those in ACD1. However, interestingly, at high log*S* range (> − 1.75), TCMCD1 (9.6%) has higher percentage than ACD1 (6.9%); moreover, at low log*S* range (<−6.0), TCMCD1 (26.7%) also has higher percentage than ACD1 (13.5%). It is obvious that TCMCD1 has a wider distribution of log*S* than ACD1.

Three descriptors, including PSA, *n*_HBD_ and *n*_HDA_, represent the electrostatic or H-bonding features of a molecule. The mean values of PSA for MDDR1, TCMCD1 and ACD1 are 88.2, 84.5 and 61.4, respectively. As shown in Figure 1, the PSA distribution of TCMCD1 is quite similar to that of MDDR1, but is slightly skewed toward higher value. The mean values of *n*_HBD_ and *n*_HBA_ are listed in Table 1 and their distributions are displayed in Figure 1. The mean values of *n*_HBD_ and *n*_HBA_ for TCMCD1 compounds are 2.12 and 5.27, respectively, which are slightly larger than those for MDDR1 compounds (1.64 and 4.83) while substantially larger than those for ACD1 compounds (1.25 and 3.22). An examination of the frequency of occurrence of elemental composition (Table 1) shows that TCMCD1 has much fewer nitrogen atoms than MDDR1 and ACD1, but has much more oxygen atoms than MDDR1 and ACD1. On average, the total number of oxygen and nitrogen atoms for ACD1, MDDR1, and TCMCD1 are 3.84, 6.01, and 5.50, respectively. Therefore, in comparison with ACD1, natural compounds in TCMCD1 have significantly more polar functions groups which can act as H-bond acceptors.

The distributions of the number of rotatable bonds for ACD1, MDDR1 and TCMCD1 are shown in Figure 1, with the mean numbers given in Table 1. All the studied datasets follow an asymmetrical Gaussian distribution. The mean numbers of rotatable bonds for ACD1, MDDR1 and TCMCD1 are 4.28, 6.45 and 4.44, respectively. Moreover, compared with ACD1, TCMCD1 is more skewed to lower values. Considering that the mean MW of TCMCD1 is significantly larger than that of ACD1, natural compounds in TCMCD1 are substantially more rigid than compounds in ACD1. It is well-known that the flexibility of a molecule is essential to determine its binding capacity in the active site of a target. In principle, when two molecules with different flexibility can form the same interaction patterns with a target, the rigid molecule usually has stronger binding affinity than the flexible one due to lower entropic loss [8]. Therefore, molecules in TCMCD1 have thermodynamic advantages to achieve more favorable binding properties than those in ACD1 and MDDR1. Our observations for TCMCD1 are also consistent with the results reported by Feher and Schmidt [8]. They found that the dataset of 3287 natural compounds has a steadily decreasing distribution from the peak to zero rotatable bonds. The prevalence of rings was believed to be another measurement for the rigidity of molecules. There are 14 descriptors in Table 1 to characterize the ring systems in molecules. As can be seen from Table 1, the mean number of bonds in aromatic rings of TCMCD1 (5.79) is obviously lower than those of MDDR1 (12.4) and ACD1 (8.16), and the mean number of bonds in ring systems (16.9) of TCMCD1 is slightly lower than that (18.6) of MDDR1 while significantly larger than that of ACD1 (10.6). Therefore, natural compounds in TCMCD1 are on average much more saturated than non-drug-like molecules in ACD1 and drug-like molecules in MDDR1. The most apparent difference between natural compounds in TCMCD1 and other molecules is the greater mean value of bonds in bridgehead ring systems (any rings that share more than one bond in common). The averaged number of bonds in bridgehead ring systems (1.75) of TCMCD1 is significantly larger than those of MDDR1 (0.456) and ACD1 (0.088). That is to say, even when the number of rings in TCMCD1 is smaller than that in MDDR1, the rings in TCMCD1 are frequently linked together to form more complicated ring systems. Certainly, the complicated ring systems of natural molecules in TCMs have positive contribution to stabilize the molecules. Moreover, the higher percentages of stereo atoms and stereo bonds (4.14 and 1.06) for natural compounds in TCMCD1, in contrast to 1.25 and 0.49 in MDDR1 and 0.55 and 0.16 in ACD1, also reflect the difference in structural complexity: natural compounds in TCMCD1 are obviously more complicated than those in MDDR1 and substantially more complicated than those in ACD1.

In summary, if all molecules with MW < 600 were included in analysis, the property distributions of TCMCD1 are remarkably different from those of ACD1, and quite similar to those of MDDR1. However, when comparing MDDR1 and TCMCD1, one still can find some apparent differences. For example, on average, TCMCD1 contains about 2 less rotatable bonds, 1.9 more oxygen atoms, 2.4 less nitrogen atoms, 6.6 less aromatic bonds, 1.3 more bonds in bridgehead ring systems, and about 2.9 more stereo atoms than MDDR1. As a comparison study, the mean values of the molecular property descriptors for the molecules with MW less than 800 (ACD2, MDDR2 and TCMCD2) were also summarized in Table 1. It is obvious that two different MW cutoffs lead to consistent conclusions. In brief, natural compounds in TCMCD2 are more complicated than drug-like molecules in MDDR2 and non-drug-like molecules in ACD2.

### Distributions of important properties for ACD3, MDDR1 and TCMCD3

**Figure 2:**
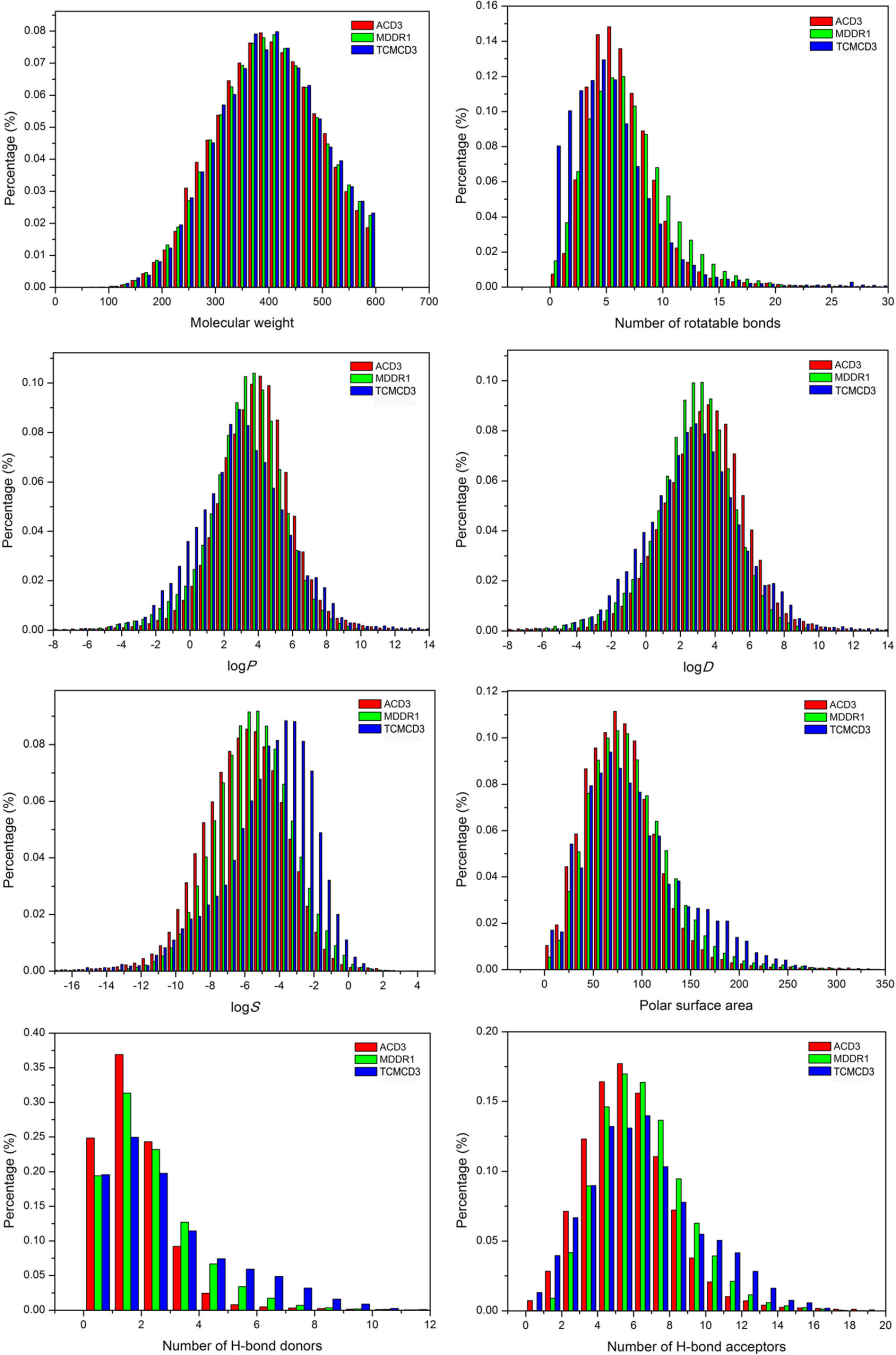
The distributions of eight important molecular property descriptors for ACD3, MDDR1 and TCMCD3.

According to the profile analysis of the property distributions in the previous section, it appears that TCMCD1 is much closer to MDDR1 than ACD1. Therefore, TCMCD may be a good source of lead or drug-like molecules. It must be noted that ACD1, MDDR1 and TCMCD1 have quite different MW distributions as shown in Figure 1. Considering that a lot of molecular properties are closely related to MW, it is possible that the distribution difference shown in Figure 1 is simply caused by the influence of MW. In order to uncover the fundamental difference of the important molecular properties among MDDR, ACD and TCMCD, we extracted two subsets from ACD1 and TCMCD1, namely, ACD3 and TCMCD3, which have similar MW distributions to MDDR1; then we performed profile analysis of the selected molecular properties for ACD3, MDDR1 and TCMCD3. As shown in Figure 2 and Table 1, the MW distributions and the mean values of MW for ACD3, MDDR1 and TCMCD3 (398, 400 and 400, respectively) are almost the same, and therefore we believe that the dependence of property distributions on MW can be effectively eliminated. The distributions of eight important molecular properties are shown in Figure 2 and the mean values for all descriptors are summarized in Table 1.

The distributions of log*P* and log*D*_7.4_ for ACD3, MDDR1 and TCMCD3 are shown in Figure 2, with the mean values listed in Table 1. Compared with TCMCD1, the mean log*P* of TCMCD3 increases slightly from 2.84 to 3.02; while the mean log*P* of ACD3 increases greatly from 2.29 to 3.84 compared with that of ACD1. Therefore, when ACD3 and MDDR1 have similar MW distributions, the average value of log*P* for non-drug-like molecules is even larger than that of drug-like molecules. Similar finding was also observed for log*D*_7.4_: the mean value of log*D*_7.4_ for ACD3 also increases from 1.78 to 2.71, while that of TCMCD3 only changes a little. That is to say, if ACD3 and MDDR1 have similar MW distributions, ACD compounds are even more hydrophobic than MDDR compounds. This finding supports our rational hypothesis that the low mean values of log*P* and log*D*_7.4_ for ACD1 are caused by much more low MW molecules in the dataset.

The distributions of calculated log*S* for the three groups of compounds are shown in Figure 2. It is noticeable that if ACD3 and MDDR1 have similar MW distributions, MDDR1 compounds are more soluble than ACD3 compounds. Obviously, the log*S* distribution of ACD is closely related to the MW distribution. This observation is understandable because MW is an important descriptor for the prediction of solubility [43]. Correlation analysis shows that MW of ACD has an obvious anti-correlation with solubility: log*S* = −0.0097 × MW-0.78 (*r* = −0.43), indicating that molecules with lower MW usually have better solubility. Therefore, when ACD3 shifts to higher MW than ACD1, the solubility, on average, becomes worse. As demonstrated in Table 1 and Figure 2, we concluded that natural compounds in TCMCD3 are more soluble than drug-like molecules in MDDR1 and non-drug-like molecules in ACD3. The higher solubility of TCMCD3 may be partially explained by the higher occurrence of oxygen atoms in TCMCD3. The mean numbers, given in Table 1, show that TCMCD3 (5.59) has more oxygen atoms than MDDR1 (3.12) and ACD3 (3.03). According to the atom additive prediction model of solubility developed in our group [43], the positive contribution of oxygen element is very significant, and even much higher than that of nitrogen element, and therefore more oxygen atoms give more favorable contributions to solubility.

The average numbers of H-bond donors and acceptors for ACD3, MDDR1 and TCMCD3 are listed in Table 1, and their distribution curves are displayed in Figure 2. The mean numbers of H-bond donors and acceptors (2.33 and 5.82) of natural compounds in TCMCD3 are the highest in contrast to ACD3 (1.24 and 4.41) and MDDR1 (1.64 and 4.83). Interestingly, the mean number of H-bond donors of ACD3 is comparable to that of ACD1, while the mean number of H-bond acceptors increases significantly from 3.22 for ACD1 to 4.41 for ACD3. However, the mean numbers of H-bond donors and acceptors for ACD3 are still lower than those for MDDR1, which are 1.64 and 4.83, respectively. In conclusion, compared with non-drug-like molecules in ACD3 and drug-like molecules in MDDR1, natural compounds in TCMCD3 have the best H-bonding capabilities, indicated by the highest mean numbers of H-bond acceptors (5.82) and H-bond donors (2.33) and marginally higher mean polar surface area (92.4). Again this observation can also be explained by the highest mean number of oxygen atoms in TCMCD3.

The percentage distributions of the number of rotatable bonds for ACD3, MDDR1 and TCMCD3 are shown in Figure 2, with the average numbers given in Table 1. For the number of rotatable bonds, the average value increases from 4.28 for ACD1 to 6.08 for ACD3, which is just slightly lower than that for MDDR1 (6.45). Meanwhile, compared with TCMCD1, the average number of rotatable bonds for TCMCD3 only slightly increases from 4.44 to 4.88. Therefore, compared with molecules in ACD3 and MDDR1, natural compounds in TCMCD3 are obviously more rigid.

In summary, when three subsets of ACD, MDDR and TCMCD (ACD3, MDDR1 and TCMCD3) have similar MW distributions, the difference of the property distributions between ACD3 and MDDR1 is not very significant compared with that between ACD1 and MDDR1 (Figures 1 and 2 and Table 1). On the other hand, the distributions of several molecular properties for TCMCD3 show substantial difference to those of ACD3 and MDDR1, and these important properties include solubility, the number of nitrogen atoms, the number of oxygen atoms, the number of halogens, the number of bridgehead atoms connecting a bridge to a ring, the number of bonds in aromatic rings, the number of bridgehead bonds connecting a bridge to a ring, the number of rotatable bonds, the number of H-bond acceptors or donors, etc. In brief, TCMCD3 compounds are slightly more hydrophilic than MDDR1 compounds and greatly more hydrophilic than ACD3 compounds, more soluble and more rigid than MDDR1 and ACD3 compounds, and have more complicated structures, especially ring systems, than MDDR1 and ACD3 compounds. Ten representative molecules with complicated structures in TCMCD3 are illustrated in Figure S1 in the supporting materials.

### If we can develop reliable simple filters to distinguish drug-like from non-drug-like and evaluate the drug-likeness of TCMCD?

According to the analysis and discussion in the previous section, we observe that the distributions of some important molecular properties for drug-like and non-drug-like molecules do not have substantial difference when the two datasets have similar MW distributions. At this point, one may raise the following question: if drug-like and non-drug-like molecules can be distinguished by using simple property-based filters. In all these drug-like filters, Lipinski’s Rule-of-Five is the most famous one [2]. However, Rule-of-Five has been proven to be not effective to distinguish drugs from non-drugs [3]. When the violation number of Rule-of-Five less or equal to 1 was defined as the cutoff of drug-likeness, 86.8% of ACD3 molecules, 88.3% of MDDR1 molecules and 85.4% of TCMCD3 were identified to be drug-like. That is to say, when drug-like, non-drug-like and natural compounds have similar MW distributions, Rule-of-Five does not have any prediction capability to distinguish drug-like from non-drug-like molecules. Opera developed a set of filters including RNG (the number of rings) and RGB (rigid bonds) for defining drug-likeness in order to overcome the limitation of Rule-of-Five. They found that 63% of ACD compounds have 0 ≤ RNG ≤ 2 and RGB ≤ 17, while 29% of ACD compounds have 3 ≤ RNG ≤ 13 and 18 ≤ RGB ≤ 56. In contrast, 61% of MDDR compounds are in the space of dimension with RNG ≥ 3 and RGB ≥ 18, and only 25% of MDDR compounds are located in the range of 0 ≤ RNG ≤ 2 and RGB ≤ 17 [3]. Here, we also applied RNG and RGB filters to evaluate the ACD, MDDR, and TCMCD databases. If the ACD1, MDDR1 and TCMCD1 subsets (MW < 600) were used, 76.3% of ACD1 molecules, 14.0% of MDDR1 molecules and 23.6% of TCMCD1 molecules have 0 ≤ RNG ≤ 2 and RGB ≤ 17, while 17.3% of ACD1 molecules, 74.3% of MDDR1 molecules and 63.1% of TCMCD1 molecules have RNG ≥ 3 and RGB ≥ 18. That is to say, most molecules in MDDR1 and TCMCD1 are drug-like while most molecules in ACD1 are non-drug-like. However, if the three subsets, ACD3, MDDR1 and TCMCD3, with similar MW distributions were used, 29.8% of ACD3 molecules, 14.0% of MDDR1 molecules and 13.6% of TCMCD3 molecules have 0 ≤ RNG ≤ 2 and RGB ≤ 17 while 59.5% of ACD3 molecules, 74.3% of MDDR1 molecules and 72.1% of TCMCD3 molecules have RNG ≥ 3 and RGB ≥ 18. That is to say, if ACD molecules have similar MW distributions to MDDR molecules, most ACD molecules (59.5%) were also identified to be drug-like. Therefore, it is obvious that the prediction accuracy of Opera’s filters for non-drug-like molecules is closely related to the MW distribution of the non-drug-like dataset. If the non-drug-like dataset has the similar MW distribution to the drug-like dataset, the false positive rate of the Opera’s filters for non-drug-like molecules is very high.

Then we evaluated the performance of 44 molecular descriptors in Additional file 1: Table S1 and 16 size-independent molecular descriptors in Additional file 1: Table S2 to distinguish MDDR1 from ACD3. The classification performance of these descriptors is summarized in Additional file 1: Tables S3 and S4 in the supplementary materials. For all these molecular descriptors, most of them do not show any classification capability, and only five of them can achieve global classification accuracy higher than 0.60. The top six descriptors for distinguish MDDR1 from ACD3 are FASA-, C3, C3P, PEOE_VAS_FPOS, PEOE_VAS_FNEG and UNC_C3. However, it is interesting to find that C3P underperforms than FASA-, and UNC_C3 performs worst in the top six descriptors. As shown in Additional file 1: Table S4, the global accuracy (GA) of FASA- is around 62.6%. According to the definition, FASA- is the fractional water accessible surface area of all atoms with negative partial charge. In a molecule, the polar atoms (such as O, S, N, etc.) usually have negative charges. If FASA- smaller than 0.339 was used as a drug-likeness filter, ~62% of MDDR1 molecules were identified to be drug-like, while only ~38% of ACD3 molecules to be drug-like. Evaluated by FASA- (<0.339), ~85.9% (29167/33961) of molecules in TCMCD3 can be identified as drug-like molecules. That is to say, the percentage of drug-like molecules in TCMCD3 is even higher than that in MDDR1. Obviously, FASA- is not a reliable filter to discriminate drug-like from non-drug-like molecules, but our analysis indicates that TCMCD may be a good source of drug-like molecules.

### Principle component plots for ACD, MDDR and TCMCD

The property distributions shown in Figure 2 and the mean values of the studied molecular descriptors summarized in Table 1 demonstrate that some property distributions of natural compounds in TCMCD are substantially different from those in ACD and MDDR even when TCMCD3, MDDR1 and ACD3 have similar MW distributions.

**Figure 3:**
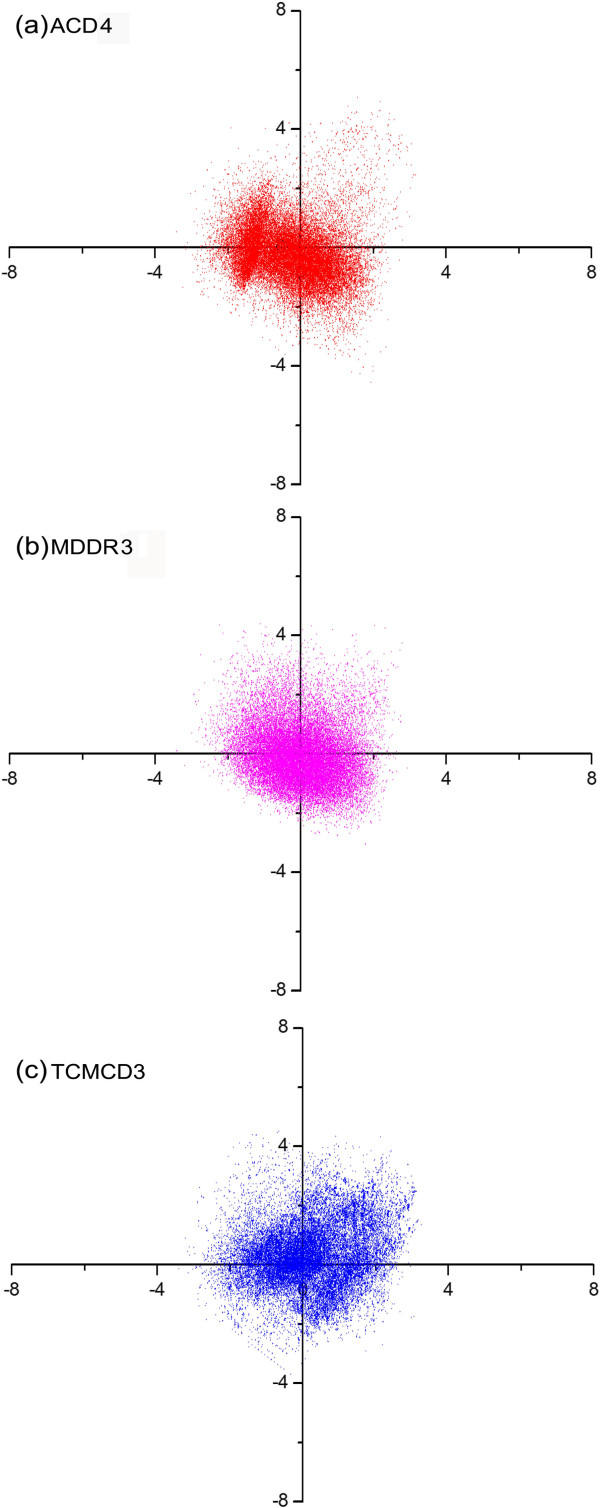
The plot of the first two principal components for (a) ACD4, (b) MDDR3 and (c) TCMCD3.

In order to characterize the spatial distribution of ACD3, MDDR1 and TCMCD3 with similar MW distributions, we conducted PCA for ACD4, MDDR3 and TCMCD3. It is pointed out that ACD4 and MDDR3 are randomly selected from ACD3 and MDDR1, respectively. All the three data sets, ACD4, MDDR3 and TCMCD3 have the same numbers of molecules (33,961) with similar MW distributions. The plot of the first two principle components is shown in Figure 3. The first two components explain about 41.9% of the variance, and the first three explain about 54.0%. In Figure 3, it is apparent that the ACD4 and MDDR3 databases cover similar space. Therefore, when the ACD and MDDR databases share similar MW distributions, the property profiles of these two databases (ACD4 and MDDR3) do not have significant difference in general. However, the distribution pattern of TCMCD3 in Figure 3 is substantially different from those of ACD4 and MDDR3. Moreover, it appears that TCMCD3 has more diverse distribution in principal component plot than ACD4 and MDDR3. Our finding is very interesting because it demonstrates that natural compounds in TCMCD3 have different and even more diverse distributions than either drug-like compounds in MDDR3 or non-drug-like compounds in ACD4 when TCMCD3, MDDR3 and ACD4 have similar MW distributions. We believe that some molecules in TCMCD can fall into the chemical space not covered by MDDR and ACD.

## Conclusion

To evaluate the drug-likeness of natural compounds from traditional Chinese medicines quantitatively, we have examined three compound collections, including ACD, MDDR and TCMCD, with respect to the distribution profiles of a variety of molecular descriptors. The comparison of property distributions for ACD1 and MDDR1 shows that the distributions of the important physicochemical properties for drug-like and non-drug-like molecules have intrinsic difference, but those for TCMCD1 are much more similar to those of MDDR1 than those of ACD1.

In order to remove the dependence of molecular weight on the analysis, we generated three subsets of ACD, MDDR and TCMCD, namely ACD3, MDDR1 and TCMCD3, with similar MW distributions and conducted the distribution analysis of the representative physicochemical properties. The analysis demonstrates that the property distributions of drug-like and non-drug-like molecules do not differ significantly anymore, implying that a single molecular property may not be used as a effective filter to distinguish drug-like from non-drug-like molecules. Compared with ACD3 and MDDR1, TCMCD3 still shows substantial difference for several molecular properties. On average, TCMCD3 compounds are a little more hydrophilic than MDDR1 compounds and substantially more hydrophobic than ACD3 compounds, more soluble and more rigid than MDDR1 and ACD3 compounds, and have more complicated structures, especially ring systems, than MDDR1 and ACD3 compounds.

Then, we quantitatively characterized the performance of each molecular descriptor to distinguish drug-like from non-drug-like molecules. In all these studied descriptors, the fractional negative accessible surface area (FASA-) outperforms the others. If FASA- was used as a drug-likeness filter, most molecules in TCMCD3 were identified to be drug-like. Finally, a PCA-based scheme was used to characterize the spatial distributions of molecular properties for the three classes of compounds. The principal component plots show that natural compounds in TCMCD3 have different and even more diverse distributions than either drug-like compounds in MDDR3 or non-drug-like compounds in ACD4 when TCMCD3, MDDR3 and ACD4 have similar MW distributions.

## Competing interests

The authors declare that they have no competing interests.

## Authors' contributions

MS and ST wrote the paper, implemented the methods and conducted the analysis with assistance from YL, QL, XX; TH and JW contributed to the paper and provided guidance. All authors read and approved the final manuscript.

## Supplementary Material

Additional file 1 **Part 1.** The protocol to generate the subsets of ACD, MDDR and TCMCD with similar molecular weight distributions; **Table S1.** The descriptions of the 44 molecular descriptors used for distribution analysis; **Table S2.** The descriptions of the 16 size-independent molecular descriptors based on the ratio of different molecular properties; **Table S3.** The performance of the 44 molecular descriptors to classify drug-like and non-drug-like molecules; **Table S4.** The performance of the 16 size-independent molecular descriptors to classify drug-like and non-drug-like molecules; **Figure S1.** Ten representative molecules with complicated structures in TCMCD3. Click here for file
