# Supplementary material for: Drug-likeness analysis of traditional Chinese medicines: 1. property distributions of drug-like compounds, non-drug-like compounds and natural compounds from traditional Chinese medicines
Source: J Cheminform. 2012 Nov 27;4:31. doi: 10.1186/1758-2946-4-31 (PMC3538521; doi:10.1186/1758-2946-4-31)
Supplement: Additional file 1 — Part 1. The protocol to generate the subsets of ACD, MDDR and TCMCD with similar molecular weight distributions; Table S1. The descriptions of the 44 molecular descriptors used for distribution analysis; Table S2. The descriptions of the 16 size-independent molecular descriptors based on the ratio of different molecular properties; Table S3. The performance of the 44 molecular descriptors to classify drug-like and non-drug-like molecules; Table S4. The performance of the 16 size-independent molecular descriptors to classify drug-like and non-drug-like molecules; Figure S1. Ten representative molecules with complicated structures in TCMCD3. [file 1758-2946-4-31-S1.doc]

**Supporting Materials**

**Part 1. The protocol to generate the subsets of ACD, MDDR and TCMCD with similar molecular weight distributions**: To get sub-datasets of ACD1 and TCMCD1 sharing similar molecular weight distributions with MDDR1, the following steps were carried out. First, the ACD1 and MDDR1 subsets were evenly split into thirty groups by molecular weight, and the number of compounds in each group was counted; then, some compounds in each ACD1 group were randomly extracted, and the number of the extracted compounds in each ACD1 group was equal to that in the corresponding MDDR1 group; finally, the extracted compounds were merged to generate ACD3. The TCMCD3 that shares similar molecular weight distribution with MDDR1 can also be generated as mentioned above.

Table S1. The descriptions of the 44 molecular property descriptors used for distribution analysis

| No. | Descriptors | Description |
| --- | --- | --- |
| 1 | Alog*P* | The log of octanol-water partition coefficient using Ghose and Crippen's method. |
| 2 | log*D*_7.4_ | The log of apparent octanol-water partition coefficient at pH=7.4 (logD) based on the Csizmadia’s method |
| 3 | log*S* | The log of intrinsic molecular solubility (logS) based on the model developed by Tetko |
| 4 | MW | Molecular weight |
| 5 | N_HBA_ | The number of hydrogen bond acceptors |
| 6 | N_HBD_ | The number of hydrogen bond bonds |
| 7 | N_rot_ | The number of rotatable bonds |
| 8 | PSA | Polar surface area |
| 9 | N_HBAL_ | The number of hydrogen bond acceptors used by Lipinski’s Rule-of-five |
| 10 | N_HBDL_ | The number of hydrogen bond donors used by Lipinski’s Rule-of-five |
| 11 | MSA | Molecular surface area |
| 12 | N_C_ | The number of carbon atoms |
| 13 | N_N_ | The number of nitrogen atoms |
| 14 | N_O_ | The number of oxygen atoms |
| 15 | N_Halogen_ | The number of halogens |
| 16 | N_Atom_ | The number of atoms |
| 17 | N_Bonds_ | The number of bonds |
| 18 | N_positive_ | The number of atoms with a positive charge |
| 19 | N_negative_ | The number of atoms with a negative charge |
| 20 | N_Spiro_ | The number of spiro atoms used as a linkage between two rings consisting of a single atom common to both. |
| 21 | N_BHA_ | The number of bridgehead atoms to connect a bridge to a ring. |
| 22 | N_Ringb_ | The number of bonds in a ring. |
| 23 | N_aromatic_ | The number of bonds in aromatic ring systems. |
| 24 | N_Bridge_ | The number of bonds in bridgehead ring systems, which are defined as any rings that share more than one bond in common. |
| 25 | N_Rings_ | The number of rings in the smallest set of smallest rings (SSSR). |
| 26 | N_AR_ | The number of aromatic rings in the smallest set of smallest rings (SSSR). |
| 27 | N_RA_ | The number of ring assemblies, which are defined as the fragments remaining when all non-ring bonds are removed from the molecule. |
| 28 | N_R3_ | The number of rings of size 3. |
| 29 | N_R4_ | The number of rings of size 4. |
| 30 | N_R5_ | The number of rings of size 5. |
| 31 | N_R6_ | The number of rings of size 6. |
| 32 | N_R7_ | The number of rings of size 7. |
| 33 | N_R8_ | The number of rings of size 8. |
| 34 | N_R9+_ | The number of rings of size 9 or larger. |
| 35 | N_Chains_ | The number of unbranched chains needed to cover all the non-ring bonds in the molecule. |
| 36 | N_ChainA_ | The number of chain assemblies, which are defined as the fragments remaining when all ring bonds are removed from the molecule. |
| 37 | N_Stereo_ | The number of stereo atoms |
| 38 | N_StereoB_ | The number of stereo bonds |
| 39 | SC0 | The number of zero-order subgraphs in the molecular graph. |
| 40 | SC1 | The number of first-order subgraphs in the molecular graph |
| 41 | SC2 | The number of second-order subgraphs in the molecular graph |
| 42 | SC3P | The number of third-order subgraphs in the molecular graph (the number of paths of length 3). |
| 43 | SC3C | The number of clusters. |
| 44 | SC3CH | The number of path/clusters. |

Table S2. The descriptions of the 16 size-independent molecular property descriptors based on the ratio of different molecular properties

| Descriptors | Description |
| --- | --- |
| *f*_PSA_ | Fractional polar surface area |
| *f*_rot_ | Fractional rotatable bonds |
| FASA+ | Fractional water accessible surface area of all atoms with positive partial charge. |
| FASA- | Fractional water accessible surface area of all atoms with negative partial charge. |
| FASA_H | Fractional water accessible surface area of all hydrophobic (|q_i_|<0.2) atoms. |
| FASA_P | Fractional water accessible surface area of all polar (|q_i_|>=0.2) atoms. |
| FCASA+ | Fractional positive charge weighted surface area, ASA+ times max { q_i_ > 0 } |
| FCASA- | Fractional negative charge weighted surface area, ASA- times max { q_i_ < 0 } |
| PEOE_VAS_FHYD | Fractional hydrophobic van der Waals surface area. This |
| PEOE_VAS_FNEG | Fractional negative van der Waals surface area. |
| PEOE_VAS_FPNEG | Fractional negative polar van der Waals surface area. |
| PEOE_VAS_FPOL | Fractional polar van der Waals surface area. |
| PEOE_VAS_FPOS | Fractional positive van der Waals surface area. |
| PEOE_VAS_FPPOS | Fractional positive polar van der Waals surface area. |
| C3P | The ratio of the number of sp3 hybridized C atoms to the number of the total heavy atoms except halogen atoms. |
| UNC_C3 | The ratio of the number of unsaturated carbon atoms to the number of sp3 carbon atoms |

Table S3. The performance of the 44 molecular property descriptors to classify drug-like molecules of MDDR1 and non-drug-like molecules of ACD3

| Descriptors | Cutoff | TP | FN | TN | FP | *SE* | *SP* | *PRE1* | *PRE2* | *GA* | *C* |
| --- | --- | --- | --- | --- | --- | --- | --- | --- | --- | --- | --- |
| Alog*P* | <=3.99 | 77022 | 60602 | 46905 | 63325 | 0.622 | 0.489 | 0.549 | 0.564 | 0.555 | 0.112 |
| log*D*_7.4_ | <=3.77 | 83734 | 56992 | 40193 | 66935 | 0.676 | 0.460 | 0.556 | 0.586 | 0.568 | 0.139 |
| log*S* | >-6.84 | 90616 | 43015 | 33311 | 80912 | 0.731 | 0.347 | 0.528 | 0.564 | 0.539 | 0.085 |
| MSA | >320 | 91495 | 43550 | 32432 | 80377 | 0.738 | 0.351 | 0.532 | 0.573 | 0.545 | 0.097 |
| PSA | >98.6 | 43249 | 89833 | 80678 | 34094 | 0.349 | 0.725 | 0.559 | 0.527 | 0.537 | 0.080 |
| *N*_C_ | >16.5 | 97199 | 44503 | 26728 | 79424 | 0.784 | 0.359 | 0.550 | 0.625 | 0.572 | 0.158 |
| *N*_N_ | >2.5 | 67741 | 76213 | 56186 | 47714 | 0.547 | 0.615 | 0.587 | 0.576 | 0.581 | 0.162 |
| *N*_O_ | >2.5 | 71455 | 56352 | 52472 | 67575 | 0.577 | 0.455 | 0.514 | 0.518 | 0.516 | 0.032 |
| *N*_Halogen_ | <=0.5 | 80957 | 66572 | 42970 | 57355 | 0.653 | 0.537 | 0.585 | 0.608 | 0.595 | 0.192 |
| *N*_Atom_ | >19.5 | 110483 | 31411 | 13444 | 92516 | 0.892 | 0.253 | 0.544 | 0.700 | 0.572 | 0.188 |
| *N*_Bonds_ | >21.5 | 108812 | 33603 | 15115 | 90324 | 0.878 | 0.271 | 0.546 | 0.690 | 0.575 | 0.188 |
| *N*_positive_ | <=0.5 | 117850 | 7760 | 6077 | 116167 | 0.951 | 0.063 | 0.504 | 0.561 | 0.507 | 0.030 |
| *N*_negative_ | <=1.5 | 123122 | 1220 | 805 | 122707 | 0.994 | 0.010 | 0.501 | 0.602 | 0.502 | 0.019 |
| *N*_Spiro_ | >0.5 | 2440 | 122729 | 121487 | 1198 | 0.020 | 0.990 | 0.671 | 0.503 | 0.505 | 0.042 |
| *N*_BHA_ | >1 | 5567 | 122530 | 118360 | 1397 | 0.045 | 0.989 | 0.799 | 0.509 | 0.517 | 0.102 |
| *N*_Ringb_ | >12.5 | 97062 | 48372 | 26865 | 75555 | 0.783 | 0.390 | 0.562 | 0.643 | 0.587 | 0.189 |
| *N*_rot_ | >8.5 | 30506 | 102655 | 93421 | 21272 | 0.246 | 0.828 | 0.589 | 0.524 | 0.537 | 0.092 |
| *N*_aromatic_ | <=17.5 | 97627 | 33099 | 26300 | 90828 | 0.788 | 0.267 | 0.518 | 0.557 | 0.527 | 0.064 |
| *N*_Bridge_ | >3 | 5567 | 122530 | 118360 | 1397 | 0.045 | 0.989 | 0.799 | 0.509 | 0.517 | 0.102 |
| *N*_Rings_ | >2.5 | 96714 | 48645 | 27213 | 75282 | 0.780 | 0.393 | 0.562 | 0.641 | 0.586 | 0.188 |
| *N*_AR_ | <=3.5 | 107805 | 19597 | 16122 | 104330 | 0.870 | 0.158 | 0.508 | 0.549 | 0.514 | 0.040 |
| *N*_RA_ | >2.5 | 62203 | 69723 | 61724 | 54204 | 0.502 | 0.563 | 0.534 | 0.530 | 0.532 | 0.065 |
| *N*_R3_ | >0.5 | 4643 | 121622 | 119284 | 2305 | 0.037 | 0.981 | 0.668 | 0.505 | 0.509 | 0.057 |
| *N*_R4_ | >0.5 | 5161 | 12182 | 118766 | 111745 | 0.042 | 0.098 | 0.044 | 0.0930 | 0.070 | -0.861 |
| *N*_R5_ | >0.5 | 74852 | 61576 | 49075 | 62351 | 0.604 | 0.497 | 0.546 | 0.556 | 0.550 | 0.101 |
| *N*_R6_ | >2.5 | 59838 | 73046 | 64089 | 50881 | 0.483 | 0.589 | 0.540 | 0.533 | 0.536 | 0.073 |
| *N*_R7_ | >0.5 | 7618 | 121592 | 116309 | 2335 | 0.061 | 0.981 | 0.765 | 0.511 | 0.521 | 0.109 |
| *N*_R8_ | >0 | 440 | 123750 | 123487 | 177 | 0.004 | 0.999 | 0.713 | 0.501 | 0.501 | 0.021 |
| *N*_R9+_ | >0 | 745 | 123582 | 123182 | 345 | 0.006 | 0.997 | 0.683 | 0.501 | 0.502 | 0.024 |
| *N*_Chains_ | >28.5 | 68684 | 79508 | 55243 | 44419 | 0.554 | 0.642 | 0.607 | 0.590 | 0.598 | 0.197 |
| *N*_ChainA_ | >11.5 | 80690 | 58752 | 43237 | 65175 | 0.651 | 0.474 | 0.553 | 0.576 | 0.563 | 0.127 |
| *N*_Stereo_ | >0.5 | 67808 | 80373 | 56119 | 43554 | 0.547 | 0.649 | 0.609 | 0.589 | 0.598 | 0.197 |
| *N*_StereoB_ | >0.5 | 43655 | 84496 | 80272 | 39431 | 0.352 | 0.682 | 0.525 | 0.513 | 0.517 | 0.036 |
| MW | >348 | 86687 | 38800 | 37240 | 85127 | 0.700 | 0.313 | 0.505 | 0.510 | 0.506 | 0.014 |
| *N*_HBA_ | >4.5 | 64341 | 70607 | 59586 | 53320 | 0.519 | 0.570 | 0.547 | 0.542 | 0.544 | 0.089 |
| *N*_HBD_ | >1.5 | 57291 | 83904 | 66636 | 40023 | 0.462 | 0.677 | 0.589 | 0.557 | 0.570 | 0.143 |
| *N*_HBAL_ | >5.5 | 67354 | 70781 | 56573 | 53146 | 0.543 | 0.571 | 0.559 | 0.556 | 0.557 | 0.115 |
| *N*_HBDL_ | >2.5 | 32304 | 106639 | 91623 | 17288 | 0.261 | 0.860 | 0.651 | 0.538 | 0.561 | 0.151 |
| SC0 | >19.5 | 110483 | 31411 | 13444 | 92516 | 0.892 | 0.253 | 0.544 | 0.700 | 0.572 | 0.188 |
| SC1 | >21.5 | 108812 | 33603 | 15115 | 90324 | 0.878 | 0.271 | 0.546 | 0.690 | 0.575 | 0.188 |
| SC2 | >30.5 | 107068 | 35662 | 16859 | 88265 | 0.864 | 0.288 | 0.548 | 0.679 | 0.576 | 0.186 |
| SC3P | >39.5 | 104841 | 39365 | 19086 | 84562 | 0.846 | 0.318 | 0.554 | 0.673 | 0.582 | 0.193 |
| SC3C | >9.5 | 72924 | 64128 | 51003 | 59799 | 0.588 | 0.517 | 0.549 | 0.557 | 0.553 | 0.106 |
| SC3CH | >0.5 | 4643 | 121622 | 119284 | 2305 | 0.037 | 0.981 | 0.668 | 0.505 | 0.509 | 0.057 |

Table S4. The performance of the 16 size-independent molecular property descriptors to classify drug-like molecules of MDDR1 and non-drug-like molecules of ACD3

| Descriptors | Cutoff | TP | FN | TN | FP | *SE* | *SP* | *PRE1* | *PRE2* | *GA* | *C* |
| --- | --- | --- | --- | --- | --- | --- | --- | --- | --- | --- | --- |
| C3 | >6.5 | 71843 | 80409 | 52084 | 43518 | 0.580 | 0.649 | 0.623 | 0.607 | 0.614 | 0.229 |
| C3P | >0.211 | 88974 | 61776 | 34953 | 62151 | 0.718 | 0.498 | 0.589 | 0.639 | 0.608 | 0.222 |
| ***f*_PSA_** | >0.269 | 39162 | 89860 | 84765 | 34067 | 0.316 | 0.725 | 0.535 | 0.515 | 0.521 | 0.045 |
| UNC_C3 | <=3.23 | 89282 | 60028 | 34645 | 63899 | 0.720 | 0.484 | 0.583 | 0.634 | 0.602 | 0.211 |
| FASA+ | >0.439 | 21114 | 107258 | 102813 | 16669 | 0.170 | 0.865 | 0.559 | 0.511 | 0.518 | 0.050 |
| FASA- | <=0.375 | 90909 | 64330 | 33018 | 59597 | 0.734 | 0.519 | 0.604 | 0.661 | 0.626 | 0.259 |
| FASA_H | <=0.704 | 40678 | 91739 | 83249 | 32188 | 0.328 | 0.740 | 0.558 | 0.524 | 0.534 | 0.075 |
| FASA_P | >0.253 | 56670 | 76314 | 67257 | 47613 | 0.457 | 0.616 | 0.543 | 0.532 | 0.537 | 0.074 |
| FCASA+ | >1.32 | 81276 | 52956 | 42651 | 70971 | 0.656 | 0.427 | 0.534 | 0.554 | 0.542 | 0.085 |
| FCASA- | <=1.68 | 77145 | 59614 | 46782 | 64313 | 0.623 | 0.481 | 0.545 | 0.560 | 0.552 | 0.105 |
| PEOE_VAS_FHYD | <=0.84 | 55257 | 74246 | 68670 | 49681 | 0.446 | 0.599 | 0.527 | 0.520 | 0.522 | 0.046 |
| PEOE_VAS_FNEG | <=0.499 | 82598 | 66817 | 41329 | 57110 | 0.667 | 0.539 | 0.591 | 0.618 | 0.603 | 0.207 |
| PEOE_VAS_FPNEG | >0.0835 | 63635 | 64662 | 60292 | 59265 | 0.513 | 0.522 | 0.518 | 0.517 | 0.518 | 0.035 |
| PEOE_VAS_FPOL | >0.205 | 34622 | 93233 | 89305 | 30694 | 0.279 | 0.752 | 0.530 | 0.511 | 0.516 | 0.036 |
| PEOE_VAS_FPOS | >0.479 | 94068 | 55537 | 29859 | 68390 | 0.759 | 0.448 | 0.579 | 0.650 | 0.604 | 0.218 |
| PEOE_VAS_FPPOS | >0.0612 | 60327 | 69537 | 63600 | 54390 | 0.487 | 0.561 | 0.526 | 0.522 | 0.524 | 0.048 |


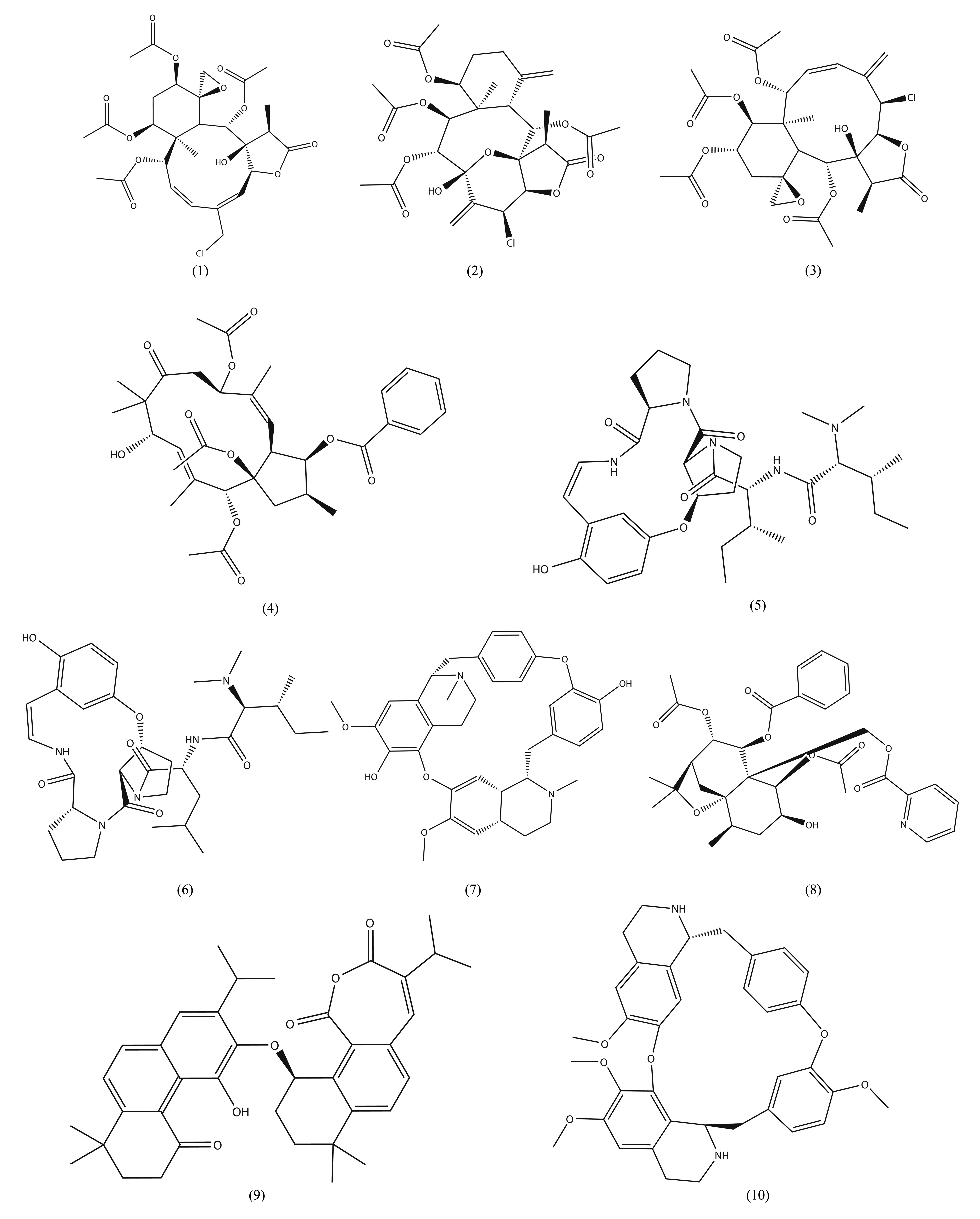


Figure S1. Ten representative molecules with complicated structures in CMCD3
